# Comparison of the Burdens and Attitudes Between Standard and Web-Based Remote Programming for Deep Brain Stimulation in Parkinson Disease: Survey Study

**DOI:** 10.2196/57503

**Published:** 2024-10-23

**Authors:** Xiaonan Wan, Zhengyu Lin, Chengcheng Duan, Zhitong Zeng, Chencheng Zhang, Dianyou Li

**Affiliations:** 1 Department of Neurosurgery, Center for Functional Neurosurgery, Ruijin Hospital, Shanghai Jiaotong University School of Medicine, No. 197 Ruijin Second Road, Shanghai (Yongjia Intersection), Shanghai, 200025, China, 86 021-64370045; 2 Clinical Neuroscience Center, Ruijin Hospital, Shanghai Jiao Tong University School of Medicine, Shanghai, China; 3 Clinical Neuroscience Center, Ruijin Hospital Luwan Branch, Shanghai Jiao Tong University School of Medicine, Shanghai, China

**Keywords:** remote programming, Parkinson disease, deep brain stimulation, telemedicine, economic evaluation

## Abstract

**Background:**

Remote programming enables physicians to adjust implantable pulse generators over the internet for patients with Parkinson disease who have undergone deep brain stimulation (DBS) surgery. Despite these technological advances, the demand for and attitudes toward remote programming compared with standard programming among patients with Parkinson disease are still not well understood.

**Objective:**

This study aims to investigate the preferences and perceptions associated with these 2 programming methods among patients with Parkinson disease through a web-based survey.

**Methods:**

A web-based survey was administered to 463 patients with Parkinson disease who have undergone DBS surgery. The survey aimed to assess the burdens associated with postoperative programming and to compare patients’ attitudes toward the 2 different programming methods.

**Results:**

A total of 225 patients completed the survey, all of whom had undergone standard programming, while 132 patients had also experienced remote programming. Among those who received standard programming, 191 (85%) patients required the support of more than 1 caregiver, 129 (58%) patients experienced over 2 days of lost work time, 98 (42%) patients incurred expenses ranging from US $42 to US $146, and 14 (6%) patients spent over US $421. Of the 132 patients who had used remote programming, 81 (62%) patients indicated a preference for remote programming in the future. However, challenges with remote programming persisted, including difficulties in obtaining official prescriptions, a lack of medical insurance coverage, and limited medical resources.

**Conclusions:**

Postoperative programming of DBS imposes significant burdens on patients and their caregivers during standard programming sessions—burdens that could be mitigated through remote programming. While patient satisfaction with remote programming is high, it is imperative for clinicians to develop personalized programming strategies tailored to the needs of different patients.

## Introduction

Deep brain stimulation (DBS) is recognized as a cost-effective, invasive treatment for patients with Parkinson disease [1]. DBS aims to regulate neural activity through controllable electrical currents generated by an implantable pulse generator (IPG) [2], which can be finely adjusted during programming sessions to optimize treatment effects [3]. However, the frequent long-distance travel required to access specialized medical centers often imposes significant costs and logistical challenges on patients [4], and may vary among different Parkinson disease subtypes [5].

Introduced as a viable alternative, internet-based remote programming first became operational in China in 2015. Its use expanded significantly during the COVID-19 pandemic, and it continues to be widely used, with over 10,000 sessions conducted annually [6,7]. Previous research has confirmed the satisfaction, necessity, and effectiveness of remote programming for patients with Parkinson disease with DBS implants [8-10]. However, most of these studies were conducted during the pandemic, when travel restrictions limited access to standard programming. With the lifting of COVID-19 restrictions, standard programming has resumed without these obstacles, prompting a reevaluation of the value of remote programming.

To explore the long-term value of remote programming, we conducted a comprehensive web-based survey focusing on patients with Parkinson disease who had received DBS. This study aimed to assess the burdens and factors associated with different programming methods and to compare patient attitudes toward each. The findings are intended to serve as an essential reference for specialized centers when making informed decisions about IPG selection prior to DBS surgery.

## Methods

### Study Design and Participants

A retrospective, cross-sectional, web-based survey was conducted from September 25, 2023, to October 15, 2023, following the CHERRIES (Checklist for Reporting Results of Internet E-Surveys) guidelines and CONSORT-EHEALTH (Consolidated Standards of Reporting Trials of Electronic and Mobile Health Applications and Online Telehealth) checklist [11,12]. A total of 463 patients are invited to participate through messages sent via WeChat (Tencent Co, Ltd), leveraging the platform’s extensive reach and accessibility. The message includes a brief overview of the study and an invitation link to the survey.

All participants met the following inclusion criteria: (1) diagnosed with Parkinson Disease and received DBS surgery at our center; (2) implanted with an IPG capable of a remote programming function, specifically from manufacturers SceneRay and PINS [13,14] (detailed information about the remote programming procedure can be found elsewhere [15]); (3) experienced at least one session of standard programming; and (4) was willing to participate in the survey.

In 2019, our center created a clinical database for patients with Parkinson disease to record the results of preoperative assessment and postoperative follow-up. The preoperative motor assessment of patients in this study was extracted from this database. The process of screening valid questionnaires and matching them with the database is provided in Multimedia Appendix 1.

### Questionnaire Design

We developed a comprehensive 49-item questionnaire divided into five sections: (1) basic information (Q1-10: including name, age, gender, etc), (2) burdens of standard programming (Q11-19: exploring transportation needs, caregiver requirements per session, etc), (3) burdens of remote programming (Q22-29: detailing caregiver involvement and workdays lost per remote session, etc), (4) satisfaction with remote programming (Q31-43: assessing factors such as ease of use and therapeutic effects on a 5-point Likert scale, from strongly disagree to strongly agree), and (5) open-ended questions (Q46-49: eliciting views on the pros and cons of both programming methods). A preliminary test of the questionnaire was conducted with 12 patients to refine its content. The English version of the questionnaire is available in Multimedia Appendix 2. The cost was converted to US dollars, based on the exchange rate of US $1≈¥7.12.

Section 4 of the questionnaire was adapted from a previously validated questionnaire on telemedicine in movement disorders [16]. This part was designed to compare remote programming with standard programming directly. Respondents were prompted to assess their perceptions of remote programming compared with standard programming, providing comparative insights that formed the basis of our analysis and discussion.

### Data Analysis

Demographic characteristics were summarized using median values, IQR, and frequencies (percentage). Group comparisons were conducted using the Mantel-Haenszel chi-aquare test for categorical data and either the 2-tailed *t* test or Wilcoxon rank-sum test for continuous data, depending on their distribution.

Ordinal logistic regression was used to identify factors influencing the burden of each standard programming session, including caregiver needs, lost working time, and travel costs. Variable selection for the regression model followed a stepwise (both forward and backward) approach. Statistical significance was set at a 2-tailed *P* value of less than .50.

Data analysis was performed using SAS (version 9.4; SAS Institute Inc), with all data stored and managed in Excel (Microsoft). Responses to open-ended questions were categorized and analyzed using a web-based tool (Weiciyun). Detailed processes for logistic regression and the analysis of open-ended questions are provided in Multimedia Appendix 3.

### Ethical Considerations

The study protocol was approved by the Ethics Committee of the Ruijin Hospital (Clinical Ethics Review [2023] No 231) and adhered to the principles of the Declaration of Helsinki. All patients were informed about the scientific purpose and their right to decline participation. Web-based informed consent was obtained at the beginning of the questionnaire.

## Results

### Overview of Participants

After excluding 7 records due to repeated submissions and 6 due to inaccurate names, 225 returned questionnaires were included in the analysis, resulting in a response rate of 49%. All patients had experienced standard programming, and 132 patients had also undergone remote programming (Table 1). Among those using remote programming, 54 (41%) had IPGs implanted by PINS, while the remaining 78 (59%) had devices from SceneRay.

The demographic profile was as follows: 105 (47%) were female, and the median distance from their residence to our center was 194 km, with an IQR of 91‐513 km. The median age was 66 years (IQR 59‐70), and the median disease duration was 11 years (IQR 8‐15). Additionally, 51 (23%) patients had attained at least a junior college education, 195 (87%) were married, and only 18 (8%) were still employed.

Using the Unified Parkinson Disease Rating Scale III, we categorized 135 patients as having the tremor-dominant subtype. Additionally, 42 patients were classified under the postural instability/gait disorder subtype, 13 were deemed to have an indeterminate subtype, and scores were unavailable for the remaining 35 patients.

**Table 1.** Characteristics of the respondents.

Characteristics		All (n=225)	TD^a^ (n=135)	PIGD^b^ (n=42)	*P* value (TD versus PIGD)
Age (year), median (IQR)		66 (59‐70)	66 (59‐69)	68 (57‐71)	.37
Sex (female), n (%)		105 (47)	63 (47)	19 (45)	.87
Disease duration (year), median (IQR)		11 (8‐15)	11 (8‐15)	11 (8‐14)	.71
Follow-up period (year), median (IQR)		2 (0‐3)	2 (0‐2)	2 (1-3)	.20
Distance (km), median (IQR)		194 (91‐513)	207 (98‐567)	152 (34‐367)	.09
**Education, n (%)**					.78
	Elementary school and below	61 (27)	37 (27)	12 (29)	
	High school and below	113 (50)	70 (51)	19 (45)	
	Junior college and above	51 (23)	29 (21)	11 (26)	
**Marital status, n (%)**					.56
	Married	195 (87)	114 (84)	37 (88)	
	Single, divorced, or widowed	30 (13)	21 (16)	5 (12)	
**Employment status, n (%)**					.36
	Employed	18 (8)	10 (7)	5 (11)	
	Unemployed, retired, or underage	207 (92)	125 (93)	37 (88)	

a TD: tremor-dominant.

b PIGD: postural instability/gait disorder.

### Comparison of Programming Burdens Between 2 Methods

**Figure 1.**
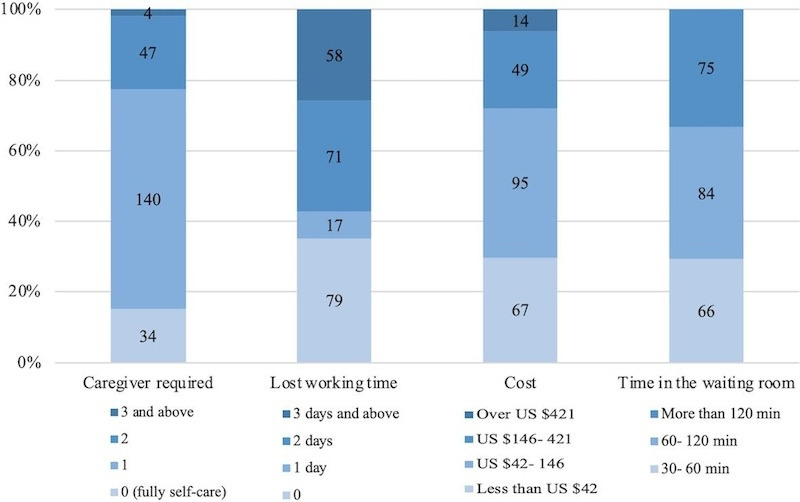
Burden of programming in standard programming with deep brain stimulation (n=225). Data are presented as numbers (center of the bar) and percentages (vertical coordinate).

For standard programming sessions (n=225), 191 (85%) patients reported requiring more than 1 caregiver for accompaniment, 129 (57%) patients indicated that more than 2 days of work were lost for themselves or their caregivers, 98 (44%) patients stated that the cost for each session ranged from US $42 to US $146, and 14 (6%) patients incurred costs exceeding US $421. Furthermore, 159 (71%) patients experienced waiting times of over 60 minutes (Figure 1). No significant differences in these burdens were observed between the tremor-dominant and postural instability/gait disorder groups (not shown).

**Figure 2.**
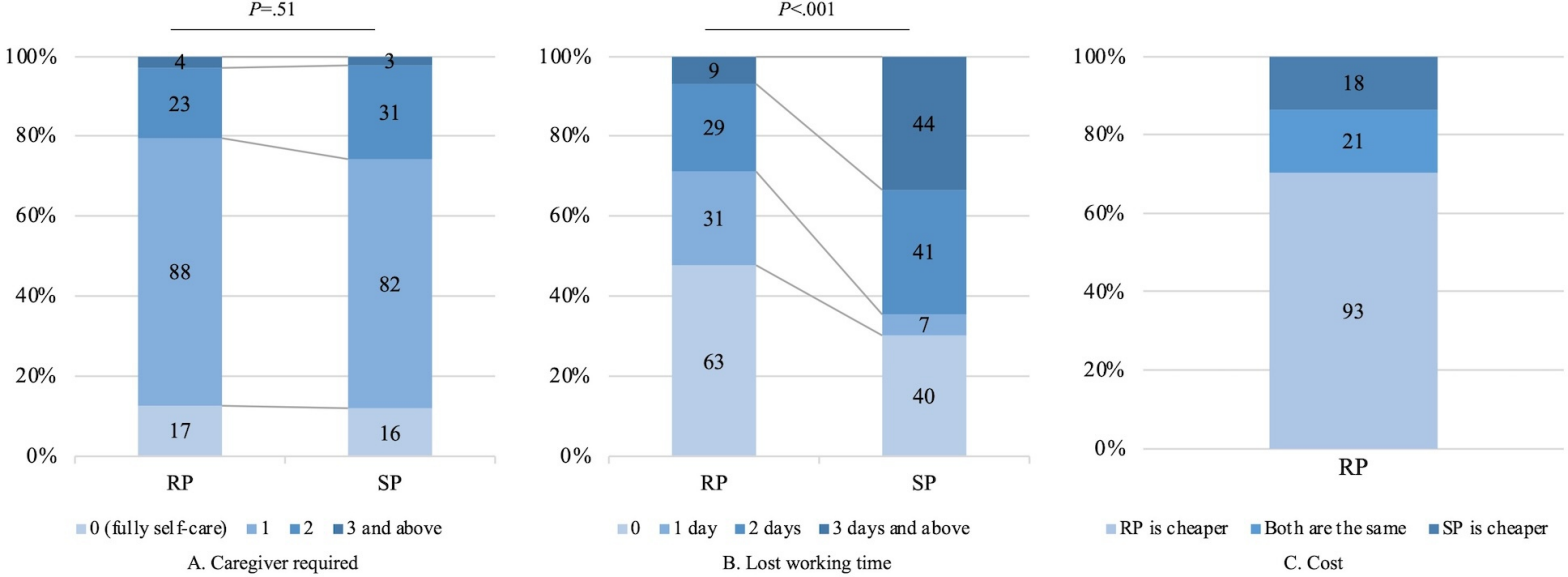
Burden between 2 programming methods (n=132). Data are presented as numbers (center of the bar) and percentages (vertical coordinate). *P*<.05 is considered statistically significant. RP: remote programming; SP: standard programming.

In remote programming sessions (n=115; Figure 2), 115 (87%) patients needed more than 1 caregiver, 38 (29%) patients reported more than 2 days of lost working time, and 93 (70%) patients perceived their costs of remote programming to be lower than standard programming. The lost working time was significantly reduced in remote programming compared with standard programming (*P*<.001), although no significant difference was noted in the number of required caregivers (*P*=.51).

### Factors Associated With Standard Programming Burdens

In the regression analysis, covariates of age, gender, distance, marital status, and education level were considered (Table 2). The requirement for caregivers was positively correlated with age (odds ratio [OR] 1.070, 95% CI 1.038‐1.103, *P*<.001) and correlated with being female (OR 0.519, 95% CI 0.300‐0.900, *P*=.02) as well as being single (OR 0.302, 95% CI 0.131‐0.695, *P*=.005). Lost working time was positively correlated with distance (OR 1.001, 95% CI 1.000‐1.001, *P*=.003) and negatively correlated with education level (high school and below: OR 0.312, 95% CI 0.153‐0.636, *P*=.001; junior college and above: OR 0.446, 95% CI 0.250‐0.796, *P*=.006). Additionally, the travel cost was also positively correlated with distance (OR 1.002, 95% CI 1.001‐1.003, *P*<.001). No significant associations were observed between the preoperative Unified Parkinson Disease Rating Scale III score and any of the burden aspects (not shown).

**Table 2.** Factors associated with burdens of standard programming.

Factors	Caregiver required		Lost working time		Travel cost	
	OR^a^ (95% CI)	*P* value	OR (95% CI)	*P* value	OR (95% CI)	*P* value
Age	1.070 (1.038‐1.103)	<.001^b^	—^c^	—	—	—
Sex (reference=male)	0.519 (0.300‐0.900)	.02^b^	—	—	—	—
Distance	—	—	1.001 (1.000‐1.001)	.003^b^	1.002 (1.001‐1.003)	<.001^b^
Marital status (reference=married)	0.302 (0.131‐0.695)	.005^b^	—	—	—	—
Education: H^d^ (reference=E^e^)	—	—	0.312 (0.153‐0.636)	.001^b^	—	—
Education: J^f^ (reference=E)	—	—	0.446 (0.250‐0.796)	.006^b^	—	—

a OR: odds ratio.

b *P*<.05 is considered statistically significant.

c Not applicable.

d H: High school and below.

e E: Elementary school and below.

f J: Junior college and above.

### Attitude Toward 2 Programming Methods

**Figure 3.**
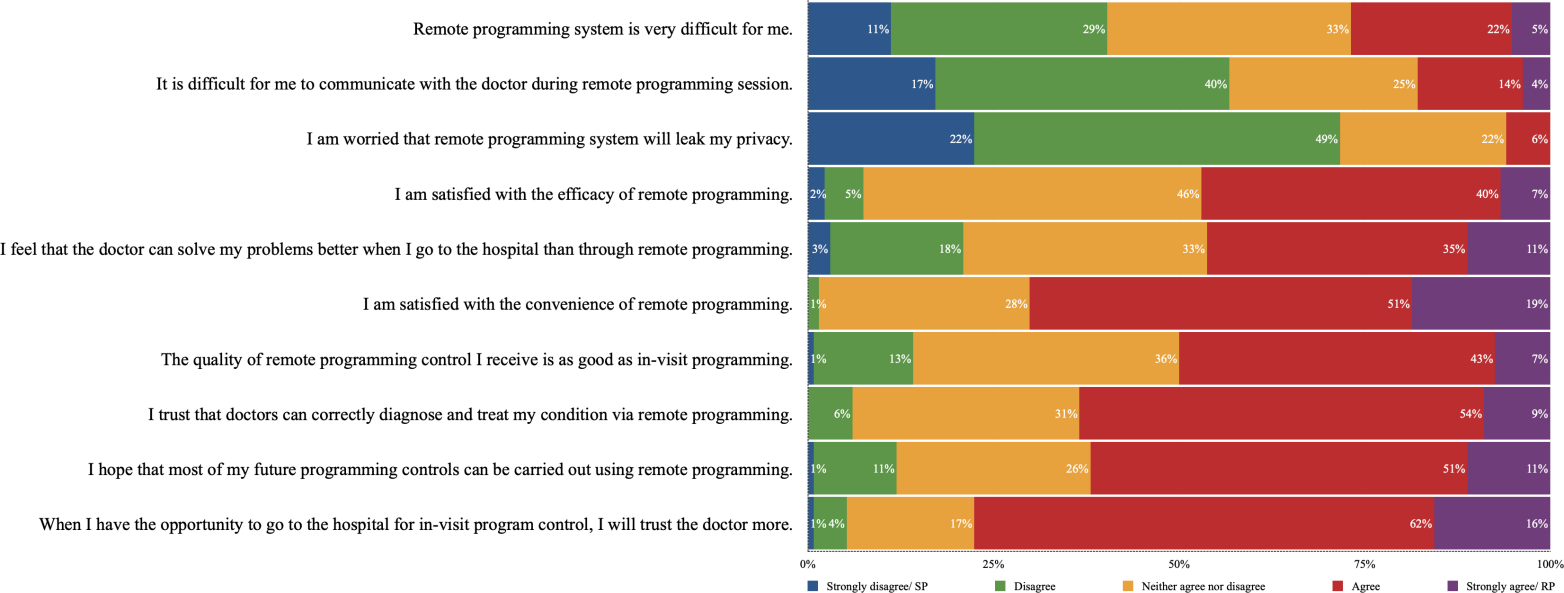
Perception of remote programming among patients (n=132). RP: remote programming; SP: standard programming.

Attitudes toward the 2 programming methods were reflected in the questionnaire results (satisfaction with remote programming section of the questionnaire). Among 132 patients with experience in remote programming (Figure 3), 53 (40%) patients disagreed (strongly) that using the remote programming system was difficult, and 75 (57%) patients disagreed (strongly) that communicating with doctors during remote sessions was challenging. A small percentage of respondents (8/132, 6%) viewed remote programming as an intrusion of their privacy, and a similar number (10/132, 7%) were (strongly) dissatisfied with the efficacy of remote programming. In addition, a significant majority appreciated the convenience (92/132, 69%) and the diagnostic accuracy (83/132, 63%) of remote programming.

When comparing remote to standard programming, 62 (47%) patients agreed (strongly) that doctors could better resolve their issues in standard programming, while 44 (33%) patients expressed a neutral opinion. Half of the patients (66/132, 50%) agreed (strongly) that the quality of remote programming matched that of standard programming. However, a substantial majority (102/132, 77%) believed their trust in doctors would increase with standard programming. Regarding their preferred method for future programming, 81 (62%) patients opted for remote programming. These results showed no significant differences between the 2 manufacturers (not shown).

Among 93 patients who had never used remote programming, those individuals typically lived closer to the medical center and had different educational levels compared with those who had used it. The top 3 reasons cited for not using remote programming were as follows: (1) a belief that remote programming might not be as convenient as hospital visits; (2) concerns about the effectiveness of remote programming; and (3) perceptions that remote programming was more expensive than hospital visits (Tables S1 and S2 in Multimedia Appendix 4).

The open-ended questions underscored the advantages and disadvantages of each programming method (Table 3). Remote programming was favored for its convenience and cost-effectiveness, though there were concerns about personal payment costs and the need for more scheduling resources. Conversely, standard programming was valued for direct doctor interaction and insurance coverage of fees, but issues like limited appointment availability and long waiting times were frequent complaints.

**Table 3.** Word frequency analysis of the pros and cons between 2 programming methods.

Programming method and category and comments ranked by frequency (top 3)			Count
**Remote programming**			
	**Neg** ^**a**^		
		Hope the cost of personal payment can be reduced.	85
		Hope there are more appointment resources.	55
		Hope the remote programming system will be easier to use.	38
	**Pos** ^**b**^		
		Remote programming is very convenient.	107
		Remote programming avoids long queue times for standard programming.	106
		Remote programming is cheaper.	40
**Standard programming**			
	**Neg**		
		It’s difficult to get an appointment for standard programming.	139
		Waiting too long in the waiting room.	117
		I hope the programming session can be longer at each time.	92
	**Pos**		
		I can communicate with the doctor face to face about my condition.	168
		The fee of programming could be paid through medical insurance.	40
		The doctor can give me a prescription directly for me to get the medication.	39

a Neg: negative comment.

b Pos: positive comment.

## Discussion

### Principal Findings

This survey study is the first to comprehensively examine the burdens associated with postoperative programming sessions of DBS from multiple perspectives. It also compares attitudes toward 2 different programming methods. Key findings include the following: (1) significant burdens were observed in terms of the number of required caregivers, lost working time, and travel costs, which heavily impacted patients and their caregivers; (2) patients facing higher risks of in-clinic programming burdens, such as those related to travel distance, age, and education level, might benefit more from remote programming; and (3) although remote programming has generally received positive feedback, there are specific areas where improvements are necessary.

As the coverage rate of DBS in standard programming increases annually [7], the associated costs are also expected to rise. A nationwide study from Hungary revealed that travel expenses could average €922.7 (≈US $1028) over 10 years following DBS [4]. Considering the median disposable income in China is US $4398 per year [17], the travel costs become significant for the 63 patients (28% of this study’s cohort) who spend more than US $421 per standard programming session, especially assuming the need for 2‐3 sessions annually post-surgery [18]. In this study, 93 (70%) patients confirmed that the costs of remote programming were lower than those of standard programming. Furthermore, a recent study indicated that, with the adoption of remote programming, 18 patients with Parkinson disease living farther from medical facilities received the same number of postoperative procedures as those residing nearby but at a lower average cost per programming session [15].

In addition to cost savings, the flexibility in time and space provided by remote programming is another substantial benefit. According to our survey, 159 (71%) patients reported needing to wait in the waiting room for more than an hour. Long waiting times coupled with short programming durations were common complaints about standard programming, as highlighted in the open-ended responses (Table 3). In China, the average waiting time for outpatient consultations in public hospitals is approximately 24.2 minutes [19]. The prolonged waiting times in DBS programming can be attributed to the time needed to observe symptom response to DBS stimulation [20], as well as the discrepancy between the high demand for postoperative programming and the limited availability of neurology specialists for programming. The web-based appointment system used in remote programming can eliminate excessive waiting times, and the home environment also provides a more comfortable and familiar setting for remote programming compared with a crowded hospital waiting room.

Patients at risk of high postoperative programming burdens are more likely to benefit from remote programming. In this study, for each standard programming session, factors such as longer distances and lower education levels were associated with increased lost working time as well as travel costs. An IPG capable of remote programming would be recommended for these patients. However, not all patients perceived remote programming to have these advantages. Ninety-three participants had never used remote programming; they believed that traditional in-person programming was a cheaper and more convenient option for them and expressed concerns about the effectiveness of remote programming. Living closer to our center may be the primary reason these individuals prefer standard programming, and varying education may also be caused by regional differences.

A previous British study highlighted that 174 (82%) patients with movement disorders preferred in-person medical treatment over telemedicine approaches once COVID-19 restrictions were lifted [16]. However, in our study, 81 (62%) patients expressed a willingness to continue using remote programming as their primary method. The contrast can be attributed to 2 factors. First, patients in this survey had undergone DBS surgery at our center and were familiar with the programming physicians, which could increase their trust in the doctor. In fact, 83 (63%) patients expressed trust in the doctor’s diagnosis, compared with only 32 (15%) patients in the previous study. Second, telephone consultations were mostly used in the previous study, while during the remote programming sessions, patients and physicians communicated through real-time videoconferencing, which may have facilitated better communication compared with the telephone.

Highlighted in the open-ended responses, remote programming was favored for its convenience and cost-effectiveness, though concerns about out-of-pocket costs and the lack of appointment resources were noted. While remote programming reduces travel expenses and is especially beneficial for patients living far from medical facilities, some still perceive it as prohibitively expensive. In China, telemedicine fees are determined by doctors based on demand and their business volume. Although most outpatient procedures are covered by medical insurance, services provided by internet hospitals typically are not, leading many patients to view these as direct expenses. Conversely, standard programming was valued for its direct communication with doctors and coverage of programming fees by insurance, but it also drew complaints regarding the availability of appointments and long waiting times (Table 3). Clinicians need to carefully consider these factors to recommend the most suitable programming method for each patient, ensuring that both medical and personal needs are effectively met. This personalized approach will help maximize the benefits of DBS therapy while minimizing its burdens.

As DBS has been increasingly used among patients with Parkinson disease, labor-intensive postoperative management is increasingly burdensome for both clinicians and patients. To enhance the programming workflow, advanced technologies like closed-loop stimulation, which relies on various input signals [21], and image-guided programming based on visualization of DBS leads [22] are being developed. Patient feedback is still indispensable in these advancements. Although indirectly, remote programming offers a convenient method for clinicians to communicate with and gather feedback from patients. The integration of remote programming with these emerging technologies holds significant potential for improving post-DBS management.

### Limitation

Several limitations of this study warrant consideration. The first is the single-center design with a low response rate. This survey was conducted at a single center and achieved a response rate of only 49%. This relatively low response rate could limit the generalizability of the findings and suggests caution when interpreting the applicability of the results to other settings. The variations in postoperative management strategies and the experience levels of programming physicians across different centers could influence the outcomes. The second limitation is the short follow-up duration. The median follow-up duration for participants in this study was 2 years. Considering that DBS has been shown to be cost-effective over a 15-year period for advanced standard programming in China [23], this limited follow-up may underestimate the long-term burdens faced by patients and their families. The third limitation is missing preoperative motor assessments. Preoperative motor assessment was not available for 32 patients, potentially compromising the validity of related conclusions. This missing data limits the strength of our findings concerning preoperative conditions and postoperative outcomes.

### Conclusion

DBS postoperative programming places burdens on patients in standard programming and their caregivers in many aspects, and remote programming could alleviate such burdens in terms of the lost working time and total costs. Identifying patients at risk of such burdens before surgery and choosing IPGs with remote programming functions would help reduce the overall postoperative burden. Although the overall satisfaction of remote programming is high, a more complete and sounder system is still required to improve postoperative management.

## Supplementary material

10.2196/57503 Multimedia Appendix 1 Data processing and cybersecurity.

10.2196/57503 Multimedia Appendix 2 Full questionnaire.

10.2196/57503 Multimedia Appendix 3 Process of logistic regression and open-ended question analysis.

10.2196/57503 Multimedia Appendix 4 Analysis for patients who never used remote programming.

## Abbreviations

CHERRIES: Checklist for Reporting Results of Internet E-Surveys
CONSORT-EHEALTH: Consolidated Standards of Reporting Trials of Electronic and Mobile Health Applications and Online Telehealth
DBS: deep brain stimulation
IPG: implantable pulse generator
OR: odds ratio
