# Supplementary material for: Comparison of the Burdens and Attitudes Between Standard and Web-Based Remote Programming for Deep Brain Stimulation in Parkinson Disease: Survey Study
Source: JMIR Aging. 2024 Oct 23;7:e57503. doi: 10.2196/57503 (PMC11523762; doi:10.2196/57503)
Supplement: Multimedia Appendix 1 [file aging-v7-e57503-s001.docx]

Multimedia Appendix 1 Data Processing and Cyber Security

Multimedia Appendix 1 illustrates the comprehensive workflow from data cleaning through to the matching process, offering a clear visual representation of each step involved.

The questionnaire is administered anonymously, with participants providing written consent before participation. Initially, the questionnaire collects basic personal identifiers such as names and dates of birth to facilitate precise matching with our existing clinical database, while ensuring that all data remains confidential.

To prevent multiple submissions by the same individual, an automated system checks the IP address of each entry. If duplicates are detected (same IP address), only the most recent submission is retained. This procedure is critical for maintaining the integrity and uniqueness of our data.

A meticulous data-matching process is employed, using key identifiers like names and dates of birth to align questionnaire responses with our clinical database. After this matching process, all personal identifying information is removed from the dataset to preserve participant anonymity.

The use of personal information is strictly limited to data matching and is promptly removed from the dataset once matching is completed. We employ robust encryption technologies for data storage and transmission to safeguard against unauthorized access. Additionally, all research data are securely stored on password-protected servers, accessible only to authorized members of the research team. Every access instance is logged in detailed audit logs, further reinforcing our commitment to data security.

Multimedia Appendix 1 Workflow of the Questionnaire Cleaning and Matching Process. Seven records were excluded due to repeated IP addresses, and six records were excluded for failing to match with the existing clinical database using patient names.
