# Supplementary material for: Comparison of the Burdens and Attitudes Between Standard and Web-Based Remote Programming for Deep Brain Stimulation in Parkinson Disease: Survey Study
Source: JMIR Aging. 2024 Oct 23;7:e57503. doi: 10.2196/57503 (PMC11523762; doi:10.2196/57503)
Supplement: Multimedia Appendix 2 [file aging-v7-e57503-s002.docx]

**Multimedia Appendix 2 Survey of Postoperative Programming for Deep Brain Stimulation in Parkinson's Disease**

Hello, we are collecting information to help improve remote programming services, aiming to improve the quality of remote programming services and save patients time and money. Your answers will not be made public and will not affect your subsequent medical services.

Section 1: Basic Information

1. Your (the patient's) name: [ Fill in the blank ] *

_________________________________

2. Please enter your (the patient's) date of birth: [ Fill in the blank ] *

_________________________________

3. Your (the patient's) gender: [ Single-choice question ] *

| ○ Male | ○ Female |  |  |  |  |  |  |
| --- | --- | --- | --- | --- | --- | --- | --- |

4. Please enter the duration of your (the patient's) illness: [ Fill in the blank ] *

_________________________________

5. Please enter your (the patient's) surgery time: [ Fill in the blank ] *

_________________________________

6. Where do you (the patient) usually live? [ Fill in the blank ] *

_________________________________

7. What is your (the patient's) educational background? [ Single-choice question ] *

| ○ Primary school |
| --- |
| ○ Middle school |
| ○ High school ( including general high school, technical secondary school, vocational high school, technical secondary school, normal school ) |
| ○ College ( including vocational college, technical college, and technical college ) |
| ○ Bachelor degree and above |

8. What is your (the patient's) marital status? [ Single-choice question ] *

| ○ Single |
| --- |
| ○ Married |
| ○ Divorced |
| ○ Widowed |

9. What is your current working status? [ Single-choice question ] *

| ○ Retirement |
| --- |
| ○ Unemployment |
| ○ Full-time employment |
| ○ Part-time employment |
| ○ Others _________________ * |

Part II: In-clinic Programming Cost Survey
This part investigates the economic burden of programming every time you return to the hospital after surgery.

10. For : In-clinic Programming, what is your (the patient's) main means of transportation to the hospital programming each time? [ Single-choice question ] *

| ○ Private car |
| --- |
| ○ Train |
| Airplane​ |
| ○ Public transportation such as subways and buses |
| ○ Others _________________ * |

11. During In-clinic Programming, which caregiver do you (the patient) need each time? [ Multiple choice ] *

| □ Completely self-sufficient |
| --- |
| □ Children |
| □ Partner |
| □ Nursing |
| □ Others _________________* |

12. During In-clinic Programming, which caregiver do you (the patient) need each time? [ Multiple choice ] *

| □ Completely self-sufficient |
| --- |
| □ Children |
| □ Partner |
| □ Nursing |
| □ Others _________________* |

13. For In-clinic Programming, how long do you (referring to the patient) and the caregiver need to take leave each time ( days ) ? [ Single-choice question ] *

| ○0 (No need to ask for leave) |
| --- |
| ○0.5 (half day) |
| ○1 |
| ○2 or more |

14. For In-clinic Programming, how many days do you (referring to the patient) need for extra accommodation each time ? [ Single-choice question ] *

| ○0 (Not required) |
| --- |
| ○1 |
| ○2 |
| ○3 or more |

15. For In-clinic Programming, how long does it usually take from the time you (the patient) plan to have the programming to the time the programming is completed ( days ) ? [ Single-choice question ] *

| ○ Less than 1 |
| --- |
| ○1 |
| ○2 |
| ○3 or more |

16. For In-clinic Programming, how long do you ( the patient) usually wait from the time you (referring to the patient) enter the waiting room to the start of programming ? [ Single-choice question ] *

| ○30 min |
| --- |
| ○60 min |
| ○120 min |
| ○ More than 120 min |

17. For In-clinic Programming, what is your (the patient's) estimate of the average cost per procedural monitoring ( including registration fees, travel, accommodation, etc. ) ( RMB ) ? [ Single-choice question ] *

| ○ Less than 300 |
| --- |
| ○300 ～ 1000 |
| ○1000 ～ 3000 |
| ○ More than 3000 |

18. For In-clinic Programming, which time period do you (the patient) want to be performed? [ Multiple choice ] [ Multiple choice ] *

| □8:00 ～ 12:00 |
| --- |
| □12:00 ～ 14:00 |
| □14:00 ～ 17:00 |
| □17:00 ～ 19:00 |
| □19:00 ～ 21:00 |

19. What is the expected time for In-clinic Programming (patient)? [ Single-choice question ] *

| ○ Monday to Friday |
| --- |
| ○ Saturday |
| ○ Sunday |
| ○ Both |

20.Have you ever used remote programming? [ Single-choice question ] *

| Yes​ |
| --- |
| ○ No |

21.Why have you never used remote programming? [ Multiple choice ] *

| □ I don’t have any remote programming equipment at home |
| --- |
| □ I think remote programming may not be effective |
| □ I think remote programming may not be as convenient as coming to the hospital for programming |
| □ I think remote programming is more expensive than going to the hospital |

Depends on option 2 of question 20

Part 3: Remote Programming Cost Survey
This part investigates the financial burden that each remote programming brings to you.

Depends on option 1 of question 20

22. For remote programming, which time period do you (the patient) want to be performed? [ Multiple choice ] [ Multiple choice ] *

| □8:00 ～ 12:00 |
| --- |
| □12:00 ～ 14:00 |
| □14:00 ～ 17:00 |
| □17:00 ～ 19:00 |
| □19:00 ～ 21:00 |

Depends on option 1 of question 20

23. What is the expected time for remote programming? [ Single-choice question ] *

| ○ Monday to Friday |
| --- |
| ○ Saturday |
| ○ Sunday |
| ○ Both |

Depends on option 1 of question 20

24. For remote programming, which caregiver do you (the patient) need for each programming? [ Multiple choice ] *

| □ Completely self-sufficient |
| --- |
| □ Children |
| □ Partner |
| □ Nursing |
|  |

Depends on option 1 of question 20

25. For remote programming, what is the highest educational level of the caregiver each time you (referring to the patient) programming the patient? [ Single-choice question ] *

| ○ Primary school |
| --- |
| ○ Middle school |
| ○ High school ( including general high school, technical secondary school, vocational high school, technical secondary school, normal school ) |
| ○ College ( including vocational college, technical college, and technical college ) |
| ○ Bachelor degree and above |

Depends on option 2, 3, 4, or 5 of question 24

26. For remote programming, how long do you (the patient) and the caregiver need to take leave each time ( days ) ? [ Single-choice question ] *

| ○0 (No need to ask for leave) |
| --- |
| ○0.5 (half day) |
| ○1 |
| ○2 or more |

Depends on option 1 of question 20

27. Do you (referring to the patient) have a sufficiently smooth and stable Internet connection at home? [ Single-choice question ] *

| Yes​ |
| --- |
| ○ No |

Depends on option 1 of question 20

28. Remote programming, where do you (referring to the patient) perform the programming each time? [ Single-choice question ] *

| ○ Living room at home |
| --- |
| ○ Bedroom at home |
| ○ Office |
| ○ Neighbor's house |
| ○ Others _________________ * |

Depends on option 1 of question 20

29. In your opinion, how does the cost of Remote programming compare to traditional face-to-face programming? [ Single Choice ] *

| ○ Remote programming costs are lower |
| --- |
| ○ Both are the same |
| ○ Remote programming costs more |

Depends on option 1 of question 20

30. For Remote programming, how long does it usually take from the time you (referring to the patient) plan to programming the patient to the time the programming is completed (days)? [ Single-choice question ] *

| ○ Less than 1 |
| --- |
| ○1 |
| ○2 |
| ○3 or more |

Depends on option 1 of question 20

Part 4: Your feelings about
remote programming The following part asks you (the patient) about your feelings and attitudes towards various issues. Please select the corresponding options

Depends on option 1 of question 20

31. Remote programming operation is difficult for me. [ Single Choice ] *

| ○ Strongly disagree |
| --- |
| ○ Disagree |
| ○ General |
| ○ Agree |
| ○ Specially agree |

Depends on option 1 of question 20

32. It is difficult for me to communicate with the doctor during Remote programming . [ Single Choice ] *

| ○ Strongly disagree |
| --- |
| ○ Disagree |
| ○ General |
| ○ Agree |
| ○ Specially agree |

Depends on option 1 of question 20

33. I am worried that Remote programming may leak my privacy. [ Single-choice question ] *

| ○ Strongly disagree |
| --- |
| ○ Disagree |
| ○ General |
| ○ Agree |
| ○ Specially agree |

Depends on option 1 of question 20

34. I am satisfied with the efficacy of Remote programming. [ Single-choice question ] *

| ○ Strongly disagree |
| --- |
| ○ Disagree |
| ○ General |
| ○ Agree |
| ○ Specially agree |

Depends on option 1 of question 20

35. I think the doctor can solve my problem better when I go to the hospital for treatment than through Remote programming. [ Single Choice ] *

| ○ Strongly disagree |
| --- |
| ○ Disagree |
| ○ General |
| ○ Agree |
| ○ Specially agree |

Depends on option 1 of question 20

36. I am satisfied with the convenience of Remote programming. [ Single Choice ] *

| ○ Strongly disagree |
| --- |
| ○ Disagree |
| ○ General |
| ○ Agree |
| ○ Specially agree |

Depends on option 1 of question 20

37. The quality of Remote programming I accept is as good as offline programming. [ Single-choice question ] *

| ○ Strongly disagree |
| --- |
| ○ Disagree |
| ○ General |
| ○ Agree |
| ○ Specially agree |

Depends on option 1 of question 20

38. I trust the doctor to correctly diagnose and treat my condition when Remote programming. [ Single Choice ] *

| ○ Strongly disagree |
| --- |
| ○ Disagree |
| ○ General |
| ○ Agree |
| ○ Specially agree |

Depends on option 1 of question 20

39. I hope that most of my future programming can be done using remote programming . [ Single Choice ] *

| ○ Strongly disagree |
| --- |
| ○ Disagree |
| ○ General |
| ○ Agree |
| ○ Specially agree |

Depends on option 1 of question 20

40. When I have the opportunity to go to the hospital for in-clinic programming, I will trust the doctor more. [ Single Choice ] *

| ○ Strongly disagree |
| --- |
| ○ Disagree |
| ○ General |
| ○ Agree |
| ○ Specially agree |

Depends on option 1 of question 20

41. In your opinion, how does Remote programming compare to traditional face-to-face programming? [ Single-choice question ] *

| ○ Remote programming is not as good as in-clinic programming |
| --- |
| ○ Remote programming is as good as in-clinic programming |
| ○ Remote programming is better than in-clinic programming |

Depends on option 1 of question 20

42. During the Remote programming, what problems do you (the patient) often encounter? [ Multiple choice ] *

| □ No failures encountered |
| --- |
| □ The internal stimulator connection fails (such as the external programmer has no power, etc.) |
| □ Unstable network signal (image and sound are stuck, delayed, unclear) |
| □ Difficulty in making reservations |
| □ Others _________________* |

Depends on option 1 of question 20

43. If you can choose your main programming method in the future, which one would you choose? [ Single-choice question ] *

| ○ Face-to-face programming |
| --- |
| ○ Remote programming |

Depends on option 1 of question 20

44.Why didn’t you choose Remote programming? [ Multiple choice ] *

| □ Can see the doctor directly |
| --- |
| □ Feel that the effect is better |
| □ I have symptoms that are difficult to describe |
| □ Lack of remote equipment |
| □ Cannot use remote system |
|  |

Depends on option 1 of question 43

45.Why choose Remote programming? [ Multiple choice ] *

| □ It is inconvenient for me to travel |
| --- |
| □ Transportation costs |
| □ No need to ask for leave |
| □ More flexible arrangements |
| □ Less waiting time |
|  |

Depends on option 2 of question 43

Part 5: Open-ended questions:
Talk about your (the patient's) suggestions and evaluations on the two programming methods.

Depends on option 1 of question 20

Part 5: Open-ended questions:
Talk about your (the patient's) suggestions and evaluations on offline programming methods

Depends on option 2 of question 20

46. What aspect of in-clinic programming do you (the patient) most hope to improve? *

Answer_________________*

Depends on option 1 of question 20

47. What is the most satisfying aspect of in-clinic programming for you (the patient)? *

Answer_________________*

Depends on option 1 of question 20

48. What aspect of Remote programming do you (the patient) most hope to improve? *

Answer_________________*

Depends on option 1 of question 20

49. What is the most satisfying aspect of Remote programming for you (the patient)? *

Answer_________________*

Depends on option 1 of question 20
