# Supplementary material for: Comparison of the Burdens and Attitudes Between Standard and Web-Based Remote Programming for Deep Brain Stimulation in Parkinson Disease: Survey Study
Source: JMIR Aging. 2024 Oct 23;7:e57503. doi: 10.2196/57503 (PMC11523762; doi:10.2196/57503)
Supplement: Multimedia Appendix 3 [file aging-v7-e57503-s003.docx]

**Multimedia Appendix 3 Process of Logistic Regression And Open-ended Question Analysis**

**1. Process of logistic regression analysis**

The software employed in this study is SAS version 9.4 (as shown in the figure below). The methodology for the logistic regression analysis presented in this article is detailed as follows: It is important to note that the code provided uses the number of caregivers as the outcome variable. The analytical approaches for other outcomes (lost working time and travel expenses), follow a similar methodology. Please be aware that the data used in the code are hypothetical and intended solely for illustrative purposes.**
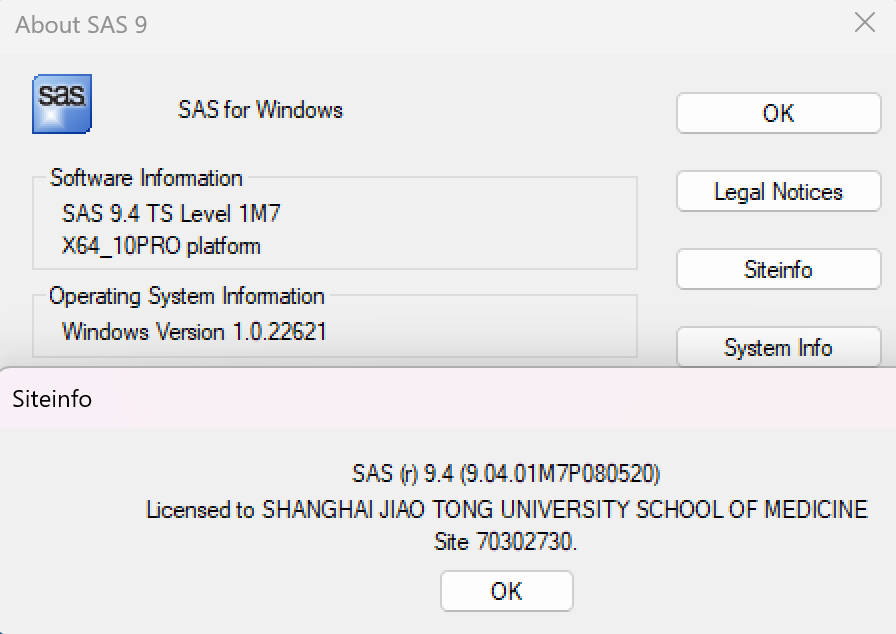
**

**Data Preparation and Variable Definition**

The analysis begins with the preparation of dataset a which contains individual-level data on various attributes. Each record in a includes variables such as id (participant identifier), age, sex, duration (duration of disease), fu (follow-up interval), dis (living distance), edu (education level), marry (marital status), job (employment status), caregiver (required caregivers), time (lost working time), and cost (travel cost). The input statement is used to read these variables, and the cards statement defines the data entries manually.

| data a;  input id age sex duration fu dis edu marry job caregiver time cost;  cards;  19 73 2 8 0 176 1 2 1 2 4 2  ...  25 45 1 2 0 697 2 1 1 0 3 1  ; |
| --- |

**Data Transformation**

In the next step, dataset b is created from dataset a to transform the education variable into binary indicators for ease of analysis. The length statement defines new variables edu1 and edu2 as length 8 bytes. Here, edu1 and edu2 are dummy variables representing different categories of the education variable (edu), created using the ifc function which generates a 1 if the condition is true, otherwise 0.

| data b;  set a;  length edu1 edu2 8.;  edu1 = ifc(edu=2, 1, 0);  edu2 = ifc(edu=3, 1, 0);  run; |
| --- |

Linear Regression Analysis

A preliminary linear regression analysis is conducted using the transformed data b. The model statement specifies the dependent variable caregiver and the independent variables. The / tol vif options are used to check multicollinearity through the tolerance and variance inflation factor metrics.

| proc reg data = b;  model caregiver= age sex duration fu dis edu1 edu2 marry job / tol vif;  quit; |
| --- |

**Logistic Regression Analyses**

Multiple logistic regression models are then fitted to assess the association between caregiver number and various predictors. The class statement specifies categorical predictors with reference coding (param=ref), where the first category is used as the reference level. The logistic models are fitted using different coding for variables and inclusion criteria:

1. The first model includes all predictors without selection to test for proportional odds.

2.The second model uses a stepwise selection method to identify significant predictors.

3.The third model applies a backward elimination method to refine the model by removing non-significant predictors.

4.The final model focuses on the final predictors of education (edu) and living distance (dis).

| proc logistic data = a descending;  class sex edu (ref=first) marry job /param=ref;  model caregiver= age sex duration fu dis edu marry job /LINK=clogit;  run;  proc logistic data = a descending;  class sex edu (ref=first) marry job /param=ref;  model caregiver= age sex duration fu dis edu marry job /stepwise details;  run;  proc logistic data = a descending;  class sex edu (ref=first) marry job /param=ref;  model caregiver= age sex duration fu dis edu marry job /backward details;  run;  proc logistic data = a descending;  class edu (ref=first) sex marry job /param=ref;  model caregiver= edu dis;  run; |
| --- |

**2. Process of open-ended question analysis**

The analysis of these open questions was conducted through several methodical steps:

1. Data Collection:

All responses from the open-ended questions were stored in Excel documents in text format for analysis.

2. Reading Through Responses and Initial Coding:

First, our team thoroughly read all responses to understand overarching themes and variations in the answers.

We then developed a coding scheme to categorize responses based on recurring themes, patterns, and frequently mentioned ideas.

3. Refining Coding:

Two staff members initially coded the data using Weiciyun software to assist in identifying preliminary themes.

A third staff member refined the coding to ensure it accurately captured the nuances of the responses, enhancing the reliability of our thematic analysis.

4. Analysis:

Finally, we analyzed the coded data to identify significant themes by examining the frequency of the codes.

We believe this addition addresses the gap mentioned and provides valuable insights into user perceptions.
