# Supplementary material for: Comparison of the Burdens and Attitudes Between Standard and Web-Based Remote Programming for Deep Brain Stimulation in Parkinson Disease: Survey Study
Source: JMIR Aging. 2024 Oct 23;7:e57503. doi: 10.2196/57503 (PMC11523762; doi:10.2196/57503)
Supplement: Multimedia Appendix 4 [file aging-v7-e57503-s004.docx]

Table S1 Characteristics between patients who received remote programming and patients who didn’t.

| Characteristics | Received RP | Not received RP | p Value |
| --- | --- | --- | --- |
| Sample size | n= 132 | n= 93 |  |
| Age- yr | 66 (58-70) | 66 (59-71) | 0.2973 |
| Sex (Female)- n (%) | 68 (51) | 39 (40) | 0.1128 |
| Disease duration- yr | 11 (8-15) | 12 (9-15) | 0.8306 |
| Follow up period- yr | 2 (0- 3) | 2 (1-3) | 0.2312 |
| Distance- km | 342 (162-848) | 98 (27-200) | <0.0001* |
| Education- n (%) |  |  | 0.0033* |
| Elementary school and below | 41 (31) | 41 (22) |  |
| High school and below | 54 (41) | 54 (63) |  |
| Junior college and above | 37 (28) | 37 (15) |  |
| Marital status- n (%) |  |  | 0.8734 |
| Married | 114 (86) | 81 (87) |  |
| Single, divorced, or widowed | 18 (14) | 12 (13) |  |
| Employment status- n (%) |  |  | 0.8262 |
| Employed | 11 (8) | 7 (8) |  |
| Unemployed, retired, or underage | 121 (92) | 86 (92) |  |

Table S2 Reasons for those who didn’t use remote programming.

| Reason (top three) | Count |
| --- | --- |
| I think remote program control may not be as convenient as coming to the hospital for program control | 42 |
| I think remote control may not be effective | 30 |
| I think remote control is more expensive than going to the hospital | 19 |
